# Monitoring spatiotemporal changes in chaperone-mediated autophagy in vivo

**DOI:** 10.1038/s41467-019-14164-4

**Published:** 2020-01-31

**Authors:** S. Dong, C. Aguirre-Hernandez, A. Scrivo, C. Eliscovich, E. Arias, J. J. Bravo-Cordero, A. M. Cuervo

**Affiliations:** 1 Department of Development and Molecular Biology, Albert Einstein College of Medicine, Bronx, NY 10461 USA; 2 Institute for Aging Studies, Albert Einstein College of Medicine, Bronx, NY 10461 USA; 3 Department of Medicine, Division of Hematology and Medical Oncology, Icahn School of Medicine at Mount Sinai, New York, 10029 NY USA; 4 The Tisch Cancer Institute, Icahn School of Medicine at Mount Sinai, New York, 10029 NY USA; 5 Department of Medicine Marion Liver Research Center, Albert Einstein College of Medicine, Bronx, 10461 NY USA

**Keywords:** Chaperone-mediated autophagy, Preclinical research

## Abstract

Autophagy malfunctioning occurs in multiple human disorders, making attractive the idea of chemically modulating it with therapeutic purposes. However, for many types of autophagy, a clear understanding of tissue-specific differences in their activity and regulation is missing because of lack of methods to monitor these processes in vivo. Chaperone-mediated autophagy (CMA) is a selective type of autophagy that until now has only been studied in vitro and not in the tissue context at single cell resolution. Here, we develop a transgenic reporter mouse that allows dynamic measurement of CMA activity in vivo using image-based procedures. We identify previously unknown spatial and temporal differences in CMA activity in multiple organs and in response to stress. We illustrate the versatility of this model for monitoring CMA in live animals, organotypic cultures and cell cultures from these mice, and provide practical examples of multiorgan response to drugs that modulate CMA.

## Abstract (web summary)

Chaperone mediated autophagy (CMA) is selective but its activity in different tissue types has been unclear due to a lack of tools. Here, the authors generate transgenic mice expressing a CMA reporter that provides spatial and temporal in vivo data, uncovering differences in CMA in distinct tissues.

## Introduction

**Fig. 1:**
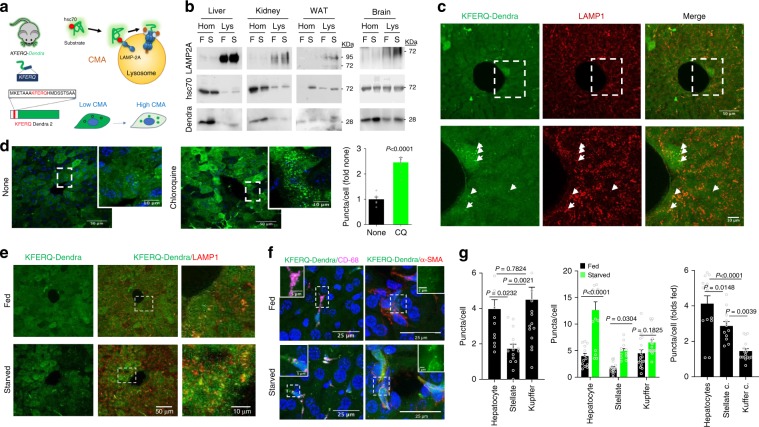
KFERQ-Dendra expression, lysosomal association and degradation in KFERQ-Dendra mice. **a** CMA-targeting motif in Dendra to generate the KFERQ-Dendra mouse lines. Right: CMA steps. **b** Representative immunoblot for Dendra in homogenates (Hom) and lysosomes (Lys) from the indicated tissues of fed (F) or 24 h starved (S) KFERQ-Dendra mice. All samples per experiment were run in the same membrane but for each protein different membranes were used. Similar results were obtained in three independent experiments. **c** Green fluorescence (from KFERQ-Dendra) and immunofluorescence (for LAMP1) of livers from 24 h starved KFERQ-Dendra mice. Bottom: Higher magnification of the dotted square.  Arrows: examples of LAMP1-positive compartments with KFERQ-Dendra. Similar results were obtained in four independent experiments. **d** Immunofluorescence for Dendra (pseudocolor in green) in liver sections from KFERQ-Dendra mice 12 h after injection with PBS (None) or chloroquine. Blue: DAPI staining. Right: Average number of fluorescent puncta in chloroquine-injected relative to PBS-injected livers. *n* = 6 (PBS) and 7 (CQ) sections (total 740 cells) coming from three independent experiments. **e, f** Immunostaining for LAMP1 (**e**) or for CD68 or α-SMA (**f**) to label Kupffer and hepatic stellate cells, respectively, in fed or 24 h starved KFERQ-Dendra mice livers. Insets: green channel at higher magnification. **g** Quantification of Dendra positive puncta in hepatocytes, Kupffer and stellate cells in fed mice livers (left), comparison in fed and starved mice livers (middle) and fold changes in Dendra-positive puncta with starvation (right). *n* = 13 (fed hepatocytes), 15 (starved hepatocytes), 17 (fed and starved Kupffer cells), 15 (fed stellate cells), and 12 (starved stellate cells) sections coming from three independent experiments. Data are presented as mean values ± s.e.m. Two-tailed unpaired *t* test (**d**), One-way ANOVA followed by Tukey’s multiple comparisons tests (**g** left and right) and Two-way ANOVA test followed by Sidak’s multiple comparisons tests (**g** middle) were used. Test statistic (*F*, *t*, *R*) and freedom degree for (**d**) and (**g**) and source data are provided in the Source Data file.

Loss of cellular proteostasis is common in old organisms and contributes to aging and age-related disorders such as neurodegenerative diseases, metabolic disorders, or cancer^1^. Autophagy is an essential component of the cellular proteostasis network by mediating degradation of organelles and proteins in lysosomes. Autophagy also contributes to cellular energetics through recycling of the essential components of the degraded material^2^. Out of the three main types of autophagy coexisting in most mammalian cells — macroautophagy, microautophagy, and chaperone-mediated autophagy (CMA) — CMA is the only one solely dedicated to protein degradation^3^. Selectivity of CMA is conferred by a dedicated protein recognition system that requires binding of the cytosolic chaperone hsc70 to a pentapeptide sequence (biochemically related to KFERQ) in the substrate protein. The hsc70/substrate protein complex is targeted to the surface of the subset of lysosomes active for CMA^3^. The substrate is transferred to the cytosolic tail of the single span lysosome-associated membrane protein type 2A (LAMP2A), triggering its assembly into a multimeric translocation complex through which the substrate protein reaches the lysosomal lumen for degradation^3^ (Fig. 1a). This unique transport mechanism and the selective targeting of substrates allows for timely removal of individual proteins through CMA^3^.

Studies in cultured cells in vitro and in rodent livers have shown that CMA is upregulated as part of the cellular response to stress including proteotoxic, genotoxic and lipotoxic stressors, oxidative stress, and hypoxia^4–7^. In some of these conditions, CMA contributes to protein quality control by eliminating damaged proteins, while in others CMA modulates changes in the activity of cellular pathways required to adapt to the stress conditions (i.e. metabolic pathways, DNA repair, etc.). CMA activity decreases with age due primarily to instability of LAMP2A in lysosomes^8^; restoration of levels of LAMP2A in liver of old mice is sufficient to improve liver proteostasis and function^9^. A primary defect in CMA has also been identified in some forms of Parkinson’s disease, frontotemporal dementia, diabetes, autoimmune disorders such as Lupus and some cancer types^3,10^.

In vitro systems with isolated lysosomes to reconstitute each of the steps of CMA have provided information on its molecular mechanisms^11^ and permitted measuring CMA in whole organs such as liver, wherein a single cell type is predominant (i.e., hepatocyte). However, measurement of CMA in intact cells was not possible until recent development and extensive validation of a fluorescent CMA reporter system^12^. In this work, we have generated a transgenic mouse model ubiquitously expressing one of these reporters (KFERQ-Dendra) and tested the suitability of using this reporter for tracking CMA in whole tissues in vivo.

The characterization of this KFERQ-Dendra transgenic mouse, reported here, demonstrates that this is a reliable experimental model to study cell type differences in basal and inducible CMA inside the same organ and to identify the spatial and temporal characteristics of CMA activity in tissues. We provide examples of the versatility of the system that allows measurement of CMA activity in organs in vivo and ex vivo, using two-photon microscopy and in vitro, using tissue slices (organotypic cultures) and primary cells isolated from organs of the KFERQ-Dendra mouse. Lastly, we provide examples to illustrate the value of this model to study changes in CMA in different tissues in response to chemical interventions in vivo. We anticipate that this model will be of great value for biomedical research to better understand the pathophysiology of CMA, and to monitor the impact of different therapeutic interventions on CMA.

## Results

### A transgenic mouse model for monitoring CMA in vivo

We have previously generated and validated a fluorescent reporter now extensively used for monitoring CMA activity in intact cells in culture^12^. The reporter, constructed by adding the KFERQ-like motif to the N-terminus of fluorescent proteins, is selectively targeted to CMA-active lysosomes by hsc70, allowing visualization of CMA activation as an increase in fluorescent puncta (Fig. 1a). Three versions of this CMA reporter using KFERQ tagged to different fluorescent proteins (photoswitchable (PS) CFP, PS-Dendra2 and photoactivable (PA) mCherry1) have been developed and extensively validated in vitro as an accurate method to measure CMA activity in transfected cells^12^. The lysosomal location of the reporter is dependent on the motif, the presence of hsc70 and LAMP2A (the CMA receptor)^12^. The amount of reporter degraded by macroautophagy is negligible (no colocalization with autophagic compartments or changes in levels when modulating macroautophagy)^12^. This reporter does not get delivered to endosomes/multivesicular bodies through endosomal microautophagy, because, contrary to CMA where this motif is necessary and sufficient, in the case of mammalian hsc70-dependent microautophagy the motif is necessary but not sufficient^13^.

To make the KFERQ-Dendra CMA reporter suitable for in vivo expression, we built a vector for donor egg injection using the pRP.ExSi plasmid backbone and inserting a region that codes for 11 amino acids including the KFERQ sequence in frame with the sequence of Dendra2 under the hybrid promoter CAGG (composed of the cytomegalovirus (CMV) immediate-early enhancer, chicken β-actin (CBA) promoter and CBA intron 1/exon 1) that provides ubiquitous and long-term expression in rodents^14^ (Supplementary Fig. 1a). Four transgenic founder mice were used to generate independent lines by crossing with wild-type mice to avoid multiple insertion sites. Because no differences were observed among lines, the final data combine all lines with similar levels of expression. We confirmed through immunoblot that KFERQ-Dendra protein was detectable in all tissues analyzed (Supplementary Fig. 1b) and that differences in tissue steady-state levels were in part due to differences in transcriptional expression across tissues (Supplementary Fig. 1c). However, for each tissue, expression of KFERQ-Dendra mRNA remained unchanged under conditions known to activate CMA such a starvation (Supplementary Fig. 1c). This was important to discard that changes in association of KFERQ-Dendra with lysosomes upon CMA activation were not a consequence of changes in the amount of protein expressed.

Isolation of lysosomes active for CMA (enriched in hsc70) from different tissues demonstrated that a fraction of the reporter reached this compartment and that, for some tissues, association of KFERQ-Dendra to lysosomes increased during starvation (Fig. 1b; note that while protein amount per line is the same, the total amount of protein per organ decreases by 40–60% resulting in lower amount of KFERQ-Dendra in all starved organs). We next confirmed that KFERQ-Dendra was degraded in vivo in lysosomes by comparing levels of the protein in tissues from mice injected or not with the protease inhibitor leupeptin to block lysosomal proteolysis under basal conditions (Supplementary Fig. 1e) or after 24 h starvation (Supplementary Fig. 1f). As expected for a CMA substrate, KFERQ-Dendra had a relatively low rate of degradation under basal conditions with the higher degradation rates in tissues such as kidney (already known to have high basal CMA activity), pancreas, heart, and white adipose tissue (WAT), which have not been previously tested for CMA (Supplementary Fig. 1g). We also observed differences in the response to starvation among organs, kidney and WAT being the most responsive tissues (Supplementary Fig. 1h). Analysis of KFERQ-Dendra in lysosomes isolated from livers and kidneys of these animals confirmed that the higher rate of degradation of KFERQ-Dendra in kidney was due to higher lysosomal uptake and not just higher proteolysis once in the lysosomal lumen, since these differences were no longer observed if the lysosomal membrane was disrupted (to allow free access of proteases to the KFERQ-Dendra protein independently of uptake) (Supplementary Fig. 1i, j).

While immunoblot allowed confirming lysosomal association and flux of the CMA substrate in different organs as a whole, it just averaged degradation rates in all cell types in the organ. To attempt visualizing CMA changes at the cellular level inside tissues, we first analyzed liver sections from starved KFERQ-Dendra mice and demonstrated that direct green Dendra-positive fluorescent puncta (emission collected at 507 nm after 488 nm excitation) colocalized with LAMP1-positive compartments, in support of lysosomal localization of the reporter in liver (Fig. 1c). As previously shown with the other CMA reporters, KFERQ-Dendra stopped fluorescing on reaching the lysosomal lumen, due to the unfolding required for translocation across the lysosomal membrane^15^ and rapid degradation in the lysosomal lumen. Thus, we found similar lysosomal association when we analyzed KFERQ-Dendra green emission or when immunofluorescence with an antibody against Dendra was performed (Supplementary Fig. 2a). The antibody signal in the red channel was not due to photoconversion of the reporter, since no signal was observed in the red channel upon capture of the Dendra fluorescent signal in slides not incubated with the antibody (Supplementary Fig. 2b). Furthermore, we confirmed that the KFERQ-Dendra bound to the surface of lysosomes at the time of isolation was internalized and readily degraded and no residual fluorescence was detected directly or with the antibody (Supplementary Fig. 2c). However, when we blocked lysosomal degradation by injecting KFERQ-Dendra mice with chloroquine, we were able to detect a significant increase (2.5 ± 0.53 folds increase) in number of fluorescent puncta per cell in liver using a Dendra antibody (Fig. 1d; mRNA levels of KFERQ-Dendra were unchanged by chloroquine under our experimental conditions Supplementary Fig. 1d). Direct fluorescence of KFERQ-Dendra at the lysosomal membrane could be used as a good read out of CMA activity, even under conditions with different lysosomal pH, as the number of KFERQ-Dendra fluorescent puncta per cell remained unchanged when pH was neutralized in cultured cells by incubation with increasing concentrations of NH_4_Cl or bafilomycin (Supplementary Fig. 2d). These findings are in agreement with substrate binding and internalization during CMA being independent of the luminal pH^16^.

These data confirm that the KFERQ-Dendra protein behaves as a typical CMA substrate in vivo, relocating from cytosol to lysosomes where it is rapidly degraded.

### Tissue-specific activation of CMA by starvation

Although, using biochemical approaches, we have extensively reported that starvation induces CMA activity in liver^17^, this mouse model allows one to separately analyze basal CMA activity and the impact of starvation in different cell types in this organ. We found that compared to hepatocytes and Kupffer cells, hepatic stellate cells have discrete levels of CMA under basal conditions (approximately half) (Fig. 1e–g). Both hepatocytes and stellate cells respond to nutrient deprivation by activating CMA whereas starvation had minimal impact on CMA activity in Kupffer cells (Fig. 1e–g). Levels of KFERQ-Dendra mRNA were comparable between hepatocytes and Kupffer cells and remained unchanged by starvation (Supplementary Fig. 2e, f), in support that changes in number of puncta per cell and KFERQ-Dendra protein levels (Supplementary Fig. 2e, g) were not due to lower transcription or synthesis but to higher degradation of the reporter protein.

**Fig. 2:**
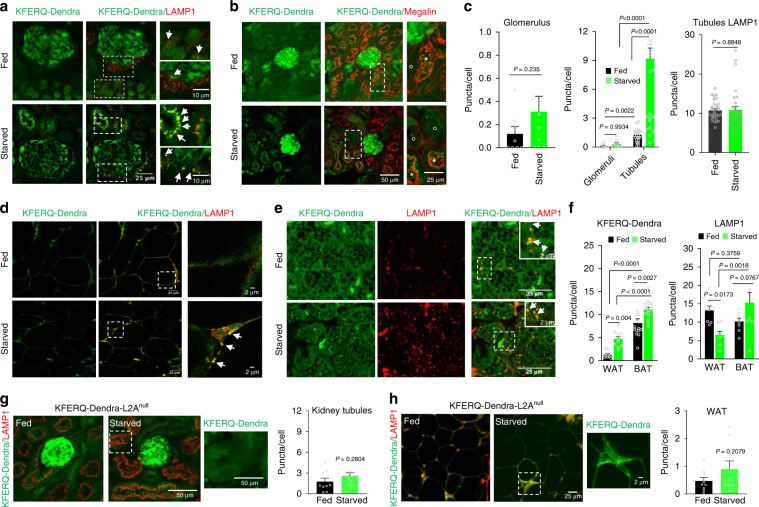
Tissue-specific changes in CMA in response to starvation. CMA (measured as number of fluorescent puncta per cell) in sections from kidney (**a**–**c**) and white and brown adipose tissue (**d**–**f**) of fed or 24 h starved KFERQ-Dendra mice. **a**, **b** Green KFERQ-Dendra fluorescence in kidney immunostained for LAMP1 (**d**) or Megalin (**e**) to stain proximal tubules. Megalin positive (star) and negative (circle) tubules and LAMP1 puncta positive for Green Dendra fluorescence (arrows) are shown. **c** Changes in Dendra-positive puncta in glomerulus (left) or glomerulus compared with tubules (middle) and in LAMP1 staining in tubules (right). Dendra values are from *n* = 8 (fed glomeruli), 9 (starved glomeruli), 27 (fed tubules) and 29 (starved tubules) sections coming from three independent experiments and LAMP1 values are from *n* = 32 (fed tubules) and 31 (starved tubules) sections from the same experiments. **d**, **e** Green KFERQ-Dendra fluorescence in white (WAT, **d**) and brown (BAT, **e**) adipose tissue immunostained with LAMP1. Arrows: LAMP1 and Green Dendra fluorescence positive puncta. **f** Dendra-positive puncta (left) and LAMP1 puncta (right) in WAT and BAT in fed and starved mice. Dendra values are from 10 (fed WAT), 11 (starved WAT), 15 (fed BAT) and 10 (starved BAT) sections coming from three independent experiments and LAMP1 values from 8 sections from all conditions from the same experiments. **g**, **h** KFERQ-Dendra fluorescence in kidney (**g**) and WAT (**h**) from fed or starved for 24 h KEFRQ-Dendra and KFERQ-Dendra-L2A^null^ mice immunostained with LAMP1. Inset: green channel of boxed area. Right: Dendra-positive puncta quantification from 9 sections of each organ and condition coming from four independent experiments. Insets show boxed areas at higher magnification. Data are presented as mean values ± s.e.m. Two-tailed unpaired *t*-tests (**c** right and left, **g**, **h**) and two-way ANOVA test followed by Sidak’s multiple comparisons tests (Fig. 2**c** middle and **f**) were used. Source data are provided in the Source Data file.

For other organs, analysis of the kidney from the KFERQ-Dendra mice demonstrated marked differences in basal CMA activity between glomeruli and tubules. Thus, the number of CMA reporter-positive puncta in tubules was almost 30 times higher than in glomeruli under basal conditions (Fig. 2a–c). We also noticed some level of heterogeneity in the number of fluorescent puncta among tubules, with proximal tubules (positive for Megalin) having the highest CMA activity (Fig. 2b and Supplementary Fig. 3a). Starvation did not result in significant changes in the number of fluorescent puncta in glomeruli, whereas tubules showed almost 5-fold increase (Fig. 2b). The increase in number of puncta was not a consequence of an expansion of the lysosomal compartment, as LAMP1 staining of tubules revealed no significant changes with starvation (Fig. 2a, c and Supplementary Fig. 3b). Overall, these studies show cell-type specific differences in basal and starvation-induced CMA activity in kidney.

Despite recent evidence of an important contribution of CMA to the regulation of lipid metabolism^18^, there is no current information on CMA activity in adipose tissue. Analysis of WAT of KFERQ-Dendra mice revealed very low basal CMA activity in this tissue but almost 5-fold increase in the number of fluorescent puncta after starvation (Fig. 2d, f). Co-staining for LAMP1 confirmed that the green fluorescent puncta were indeed lysosomes and that changes in CMA activity with starvation were independent of the number of lysosomes that if anything was reduced by starvation (Fig. 2d, f and Supplementary Fig. 3c). Similar analysis of a functionally different adipose tissue, brown adipose tissue (BAT) demonstrated very different CMA response to starvation. Thus, we found 10 times higher basal CMA activity in BAT when compared to WAT but only a discrete increase in fluorescent puncta in this tissue upon starvation (Fig. 2e, f).

Crossing KFERQ-Dendra mice with LAMP2A^null^ mice that are unable to perform CMA^19^ allowed us to further validate the selectivity of the reporter mouse model for CMA. Kidney and WAT from KFERQ-Dendra-L2A^null^ mice displayed very low number of fluorescent puncta that remained unchanged upon starvation (Fig. 2g, h), in support that association of KFERQ-Dendra with lysosomes under these conditions requires an active CMA. Furthermore, to test the robustness of the model and increase its versatility, we also changed the KFERQ-Dendra reporter from the original FVB background to a C57BL/6 background and found that differences in CMA across tissues and cell-type specific responses of CMA to starvation were conserved across strains. Similar to the FVB model, starvation activated CMA activity in hepatocytes, WAT and kidney tubular cells but not in kidney glomeruli in C57BL/6 KFERQ-Dendra mice (Supplementary Fig. 3d).

Overall, a major advantage of using the KFERQ-Dendra mouse model is that it allows analyzing cell-type specific differences in basal and inducible CMA activity, which can be of great importance in organs of complex cellularity.

### Versatility of the KFERQ-Dendra mouse to study CMA

**Fig. 3:**
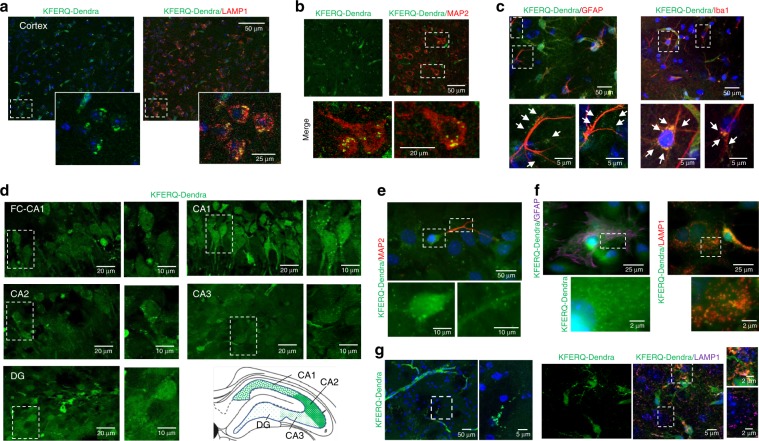
CMA activity in brain. **a** Green fluorescence (for KFERQ-Dendra) and immunofluorescence (for LAMP1) of cerebral cortex from KFERQ-Dendra mice. Insets: boxed area at higher magnification. Nuclei are highlighted in blue. **b**, **c** Green KFERQ-Dendra fluorescence in the same regions immunostained with MAP2 (to label neurons) (**b**), GFAP (to label astrocytes) or IBA1 (to label microglia). Arrows: green fluorescent puncta. Boxed areas are shown at higher magnification at the bottom (**c**). **d** Green KFERQ-Dendra fluorescence in the indicated regions of the hippocampus of KFERQ-Dendra mice. Boxed areas are shown at higher magnification on the right. The cartoon depicts the areas analyzed and abundance of the fluorescent puncta in each region. **e** Direct KFERQ-Dendra fluorescence in primary neuronal cultures prepared from KFERQ-Dendra mice brains. Insets show the boxed regions of soma and neurites at higher magnification for the green channel only to better appreciate presence of fluorescent puncta. **f** Green fluorescence (for KFERQ-Dendra) and immunofluorescence (for GFAP or LAMP1) in primary astrocyte cultures prepared from KFERQ-Dendra mice brains. Boxed areas are shown at higher magnification at the bottom. **g** Green fluorescence (for KFERQ-Dendra) and immunofluorescence (for LAMP1) in brain sections from KFERQ-Dendra mice 4 days after culture. Merged images are shown on the right, where nuclei are highlighted with DAPI. Boxed areas are shown at higher magnification on the right. Experiments were repeated 3 times (for **a**, **b**, **d**) and 5 times (for **c**, **e**, **f**, **g**) with similar results.

Although failure of CMA has been linked to several common neurodegenerative disorders such as Parkinson’s disease or frontotemporal dementia^10^, biochemical approaches have offered little information about CMA activity in the brain. Using the KFERQ-Dendra mice, we were able to detect fluorescent puncta associated to lysosomes (positive for LAMP1, Fig. 3a) in cortical sections and assign the presence of puncta to neurons (Fig. 3b) or different type of glial cells by using cell-type specific markers (Fig. 3c). These results support the suitability of this model to study differences in CMA activity among different brain cell types. Interestingly, even when considering puncta only in neurons, we detected differences in neuronal CMA activity depending on the brain region. Thus, analysis of hippocampus from KFERQ-Dendra mice revealed maximal basal CMA activity in neurons in the CA2 and CA1 regions and lower activity in the dentate gyrus neurons (Fig. 3d).

This animal model can also be used as a source for primary cells, which are more difficult to transfect and in which transduction with different lentivirus for delivery of fluorescent reporters can have an associated response to the lentivirus itself that could mask the normal physiology of the cell of interest. We have generated primary neuronal and astrocyte cultures (Fig. 3e, f) as well as organotypic brain slices cultures (Fig. 3g) from the KFERQ-Dendra mice. As in the in vivo studies, we confirmed that the fluorescent puncta in both systems colocalize with lysosomal markers (Fig. 3f, g) supporting that primary cells and tissue slices can be used ex vivo to monitor changes in CMA activity after interventions of interest. In fact, using primary cultures of astrocytes isolated from these animals, we confirmed that chemical or genetic blockage of macroautophagy does not inhibit delivery of KFERQ-Dendra to lysosomes, further supporting the selectivity of this reporter for CMA (Supplementary Fig. 4a; staining for LC3 and LAMP1 are shown as positive control for macroautophagy inhibition and absence of major disturbances in the endolysosome system, respectively). As described before in cell lines^20^, primary astrocytes respond to macroautophagy blockage by upregulating CMA, here detected as increase in KFERQ-Dendra puncta (Supplementary Fig. 4a).

In studies with cell lines transduced with CMA fluorescent reporters, photoswitching has been necessary to differentiate the fluorescence of the protein associated with lysosomes against the cytosolic fluorescent protein^12^. When using tissues and cells from KFERQ-Dendra mice, we found that at any given time, the amount of cytosolic protein was considerably lower than in transduced cells and allowed for detection of the bright lysosomal puncta without the need of photoswitching (as shown in all direct fluorescence images in this report). However, the photoconversion feature of the KFERQ-Dendra mouse, can become useful in those instances in which CMA of two different pools of proteins (i.e. before or after an intervention) needs to be tracked. As example, we exposed primary cultures of astrocytes from KFERQ-Dendra mice to paraquat (PQ) to induce oxidative stress (known activator for CMA) and by changing the time at which photoswitching was done, we could compare CMA activity during the first and last 12 h of the 24 h treatment (Supplementary Fig. 4b). As expected, CMA activity was higher in the presence of PQ, but its rates continued to increase during the whole 24 h of PQ exposure, since the amount of red fluorescent puncta observed in the last 12 h was significantly higher than in the first 12 h (Supplementary Fig. 4b). Although puncta could also be observed in the green channel, detection was less accurate because of the changes in overall cytosolic signal with the prolonged exposure to PQ.

**Fig. 4:**
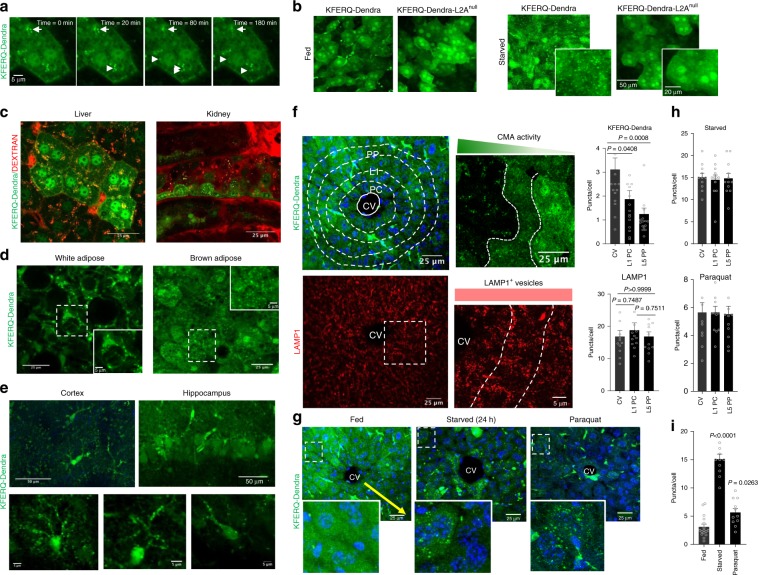
Spatial and temporal analysis of CMA. **a** Still images from an intravital imaging time-lapse movie of liver from KFERQ-Dendra mice showing the dynamics of several CMA lysosomes (arrows). Similar results were obtained in four independent experiments. **b** Representative two-photon microscopy images of livers from fed or 24 h starved KEFRQ-Dendra and KFERQ-Dendra-L2A^null^ mice. Insets: higher magnification. Similar results were obtained in three independent experiments. **c**, **d** Representative images of liver and kidney (**c**) and WAT and BAT (**d**) from 24 h starved KFERQ-Dendra mice. In **c** mice were injected with 155 KDa TRITC Dextran (red) to highlight lysosomal compartments. Similar results were obtained in four independent experiments, Insets: Boxed area at higher magnification. **e** Top: Maximum projection of cortical (left) and hippocampus (right) regions from KFERQ-Dendra mice imaged with two-photon microscopy. Bottom: examples of magnified individual cells. Similar results were obtained in four independent experiments. **f** Left top: KFERQ-Dendra mice liver section with delineated sub-zoning. CV: central vein, L1 PC: layer 1 pericentral, L5 PP: layer 5 periportal. DAPI is shown in blue. Right: Higher magnification liver region. Bottom: LAMP1 staining of full field (left) and higher magnification region with delineated zones (right) of liver. Right: Number of KFERQ-Dendra (top) and LAMP1 (bottom) positive puncta in liver regions. Values are from 16 (Dendra) and 12 (LAMP1) sections coming from 4 independent experiments. **g** Image of livers from KFERQ-Dendra mice fed, starved for 24 h or paraquat (PQ) injected. Insets: higher magnification of regions distal from the CV. Yellow arrow: KFERQ-Dendra positive puncta gradation. **h** Fluorescent puncta per cell in regions of starved or PQ-treated KFERQ-Dendra mice livers. **i** Overall number of fluorescent puncta in the same livers as (**g**). Values in **h** and **i** are from 11 (CV), 12 (L1PC) and 12 (L5PP) sections (panel **h**), and 16 (fed), 11 (starved) and 11 (PQ) sections (panel **i**) Data are presented as mean values ± s.e.m. One-way ANOVA test followed by Dunnett’s multiple comparisons test was used. Source data are provided in the Source Data file.

One additional advantage of the use of the fluorescent reporter transgenic mouse model is that it allows one to detect CMA activity directly in living animals using intravital two-photon microscopy^21,22^. These procedures allow for time-course analysis and study of changes in CMA in real time in different organs (as shown in Fig. 4a for liver). We confirmed that the fluorescent puncta observed when analyzing the intact organ with two-photon microscopy was also specific for CMA, since it was no longer detectable in livers from the KFERQ-Dendra-L2A^null^ mice even after 24 h starvation (Fig. 4b). For in vivo studies, injection of fluorescent dextran (TRITC), internalized from the bloodstream by endocytosis, can be used to highlight lysosomal compartments thus confirming that the observed KFERQ-Dendra-positive puncta were indeed lysosomes (liver and kidney are shown in Fig. 4c). Two-photon imaging can be applied to image WAT and BAT tissue (Fig. 4d). In the case of the brain, we used two-photon brain imaging along with 3D reconstructions to track CMA in cortex. KFERQ-Dendra-positive puncta was observed both in cell bodies and in all their projections, which often extend to distant brain regions (Fig. 4e, Supplementary Fig. 4c, d and Supplementary videos 1-3). For deeper areas of the brain such as hippocampus, two-photon imaging and 3D reconstruction can be used in brain slices from these mice to obtain similar information (Fig. 4e, Supplementary Fig. 4e and Supplementary video 4). This whole battery of approaches should be of use in the future to study cell-type specific changes in CMA activity in the brain in response to different stressors in real time. In addition, crossing of the KFERQ-Dendra mice with different experimental disease mouse models will allow monitoring time-course and region-specificity of changes in CMA during disease onset and progression.

Similar 3D reconstruction analysis of two-photon images from the KFERQ-Dendra mice also allowed to confirm an increase in the number of fluorescent puncta in response to starvation in organs such as the kidney (Supplementary video 5) and liver (Supplementary Fig. 5a and Supplementary video 6). This type of analysis led us to identify a zonal gradation in basal CMA activity in the liver. We found that the well-established histological liver zonation from the central vein to the portal region associates with a gradation in CMA activity, whereby pericentral regions (those with lower oxygen and nutrient flow) display higher number of KFERQ-Dendra puncta than periportal regions (Fig. 4f, and Supplementary Fig. 5b). Labeling with LAMP1 did not display such a gradation (Fig. 4f and Supplementary Fig 5c, d), thus confirming that the decrease in number of CMA reporter-positive fluorescent puncta was not due to mere decrease in the number of lysosomes in the periportal area but was indeed a manifestation of lower fraction of lysosomes being active for CMA in those regions (Supplementary Fig. 5d immunostaining shows that only a small fraction of LAMP1-positive compartments are also positive for the CMA reporter in that region). Fluorescence In Situ Hybridization (FISH) revealed that transcriptional expression of KFERQ-Dendra was homogenous across the liver lobule (Supplementary Fig. 5e, f), further suggesting that the zonal gradation in basal CMA activity was determined by changes at the level of protein degradation. Interestingly, stimuli known to activate CMA in liver, such as complete starvation^17^ or oxidative stress (induced by PQ injection in vivo)^4^ led to significant increase in number of KFERQ-Dendra puncta per cell, that was now broadly detectable through all liver regions (Fig. 4g–i and Supplementary Fig. 5a). These results further support that nutrient and oxygen gradients from the central vein to the portal area may be responsible for the zonal differences in CMA activity in liver under basal conditions, as these differences are abolished when the gradient is disrupted by complete absence of nutrients. Detecting this previously unknown spatial differences in CMA activity in liver has only been possible through use of the KFERQ-Dendra mouse model and the type of image-based analysis presented in this study.

### Practical examples of the analysis of CMA activity in vivo

We include here applied examples of the use of the KFERQ-Dendra mouse model to study changes in CMA in response to different interventions in vivo.

**Fig. 5:**
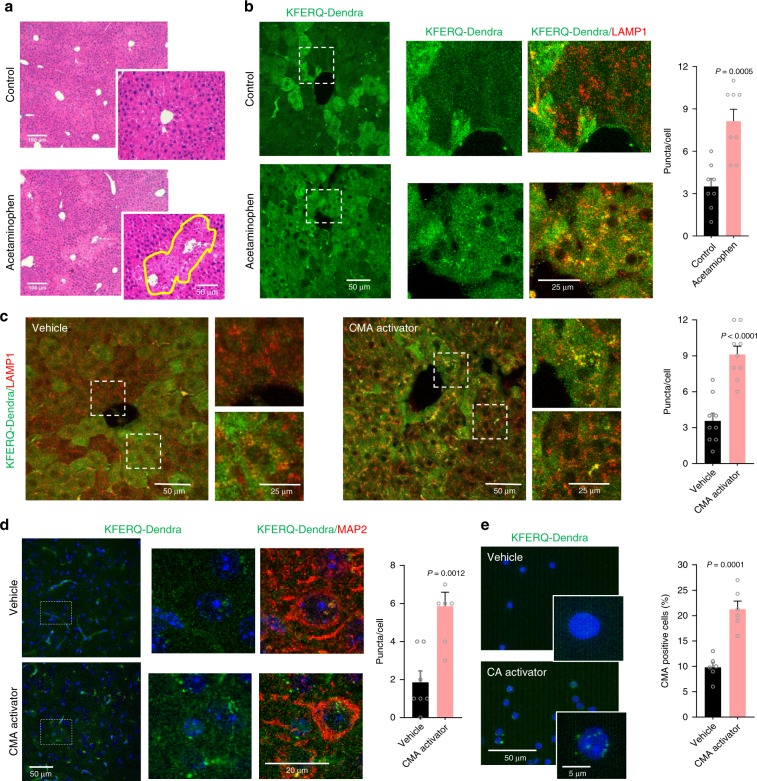
Monitoring chemical activation of CMA in vivo. **a**, **b** Livers from KFERQ-Dendra mice injected with vehicle or acetaminophen (to induce hepatotoxicity) were stained with H&E (**a**) or imaged for direct fluorescence (for KFERQ-Dendra) or immunostained (for LAMP1) (**b**). Boxed regions are shown at higher magnification on the right. Right: quantification of the number of puncta per cell. Similar results were obtained in three independent experiments for each condition for **a** and **b**. Data points indicate average values from different sections of three independent experiments (*n* = 8 sections). **c**–**e**. KFERQ-Dendra mice were i.p. injected with the CMA activator or vehicle for 3 days and different organs imaged for direct fluorescence (for KFERQ-Dendra) and immunofluorescence for LAMP1 in livers (**c**) and for MAP2 in brain cortex (**d**) and direct fluorescence in T cells (**e**) isolated from blood of the treated animals. Quantification of the average number of puncta per cell is shown on the right. Experiments were repeated three times independently for (**c**–**e**). Data points indicate average values from different sections of three independent experiments for each condition *n* = 9 sections for (**c**), *n* = 7 sections for (**d**), *n* = 6 sections for (**e**). Two-tailed unpaired *t*-tests were used.Source data are provided as a Source Data file.

We have recently found that hepatocytes defective for CMA display high sensitivity to acetaminophen, a drug commonly used for pain and fever management but that causes acute liver injury upon overdose ^23^. This finding let us to speculate that activation of CMA could be part of the front-line defense against acetaminophen-induced toxicity. To test if that was the case, we injected KFERQ-Dendra mice with vehicle or acetaminophen under conditions that we confirmed resulted in some level of hepatic damage (Fig. 5a). Acetaminophen exposure did not change expression levels of KFERQ-Dendra determined by qPCR (a highly advisable practice when introducing any new manipulation to this reporter mouse model) (Supplementary Fig. 1d), whereas fluorescence imaging of the same livers revealed a significant increase in the number of KFERQ-Dendra fluorescent puncta, thus confirming activation of CMA in response to the toxic insult (Fig. 5b).

We have also used the KFERQ-Dendra mouse model to monitor the efficiency of recently developed CMA activators in vivo^24^. Medicinal chemistry on these compounds has improved their pharmacokinetic properties making them now compatible with in vivo use. As shown in Fig. 5c, administration of single dose of the CMA activator resulted in a 2.5-fold increase in the amount of fluorescent puncta in liver. A similar increase was also observed in brain (cortex region shown here) where the number of fluorescent puncta in neurons (immunostained with MAP2) was 3-fold of that observed in vehicle-treated animals (Fig. 5d). We have also found that in the case of interventions of this type expected to result in systemic activation of CMA, the efficacy of the intervention can be monitored at different times by drawing blood from these mice and measuring changes in CMA in peripheral blood cells. For example, in KFERQ-Dendra mice treated with the CMA activators, we found a comparable increase in the number of fluorescent puncta in circulating T cells (Fig. 5e).

## Discussion

In this report, we have characterized the transgenic mouse model systemically expressing a CMA reporter and validated its use for systemic monitoring of cell and tissue-specific spatiotemporal changes in CMA. Previously used biochemical approaches to measure CMA in animal tissues, failed to discriminate cell type-dependent differences in CMA inside tissues^11^. In fact, as shown in this report, while immunoblot analysis revealed kidney as one of the tissues with upregulation of CMA in response to starvation (Supplementary Fig. 1e–g), image-based analysis in kidneys from the same KFERQ-Dendra mouse model revealed marked differences between CMA in tubules and glomeruli, whereby starvation did not have a noticeable effect in glomerular cells’ CMA (Fig. 2a–c). Findings like this highlight the potential value of this mouse model to study CMA in organs of complex cellularity. The separate analysis of CMA activity in different cell types made possible through the image-based procedures applied to this mouse model, demonstrates that cell-type specific changes in CMA activity are highly consistent across mice and even for different mice strains (Supplementary Fig. 3d).

Interestingly, even for organs such as the liver, where hepatocytes represent more than 80% of cells, the KFERQ-Dendra mouse model has permitted us to identify striking zonal differences in CMA activity (Fig. 4f, g). Far from a mere histological concept, hepatic zonation has been shown to have also important functional implications and even determine differences in regional susceptibility to different diseases^25^. It is attractive to think that differences in basal CMA activity among hepatocytes located in different zones may contribute to their vulnerability to disease. Study of these and other related questions can now be possible by combining disease-inducing interventions in the KFERQ-Dendra mouse model and intravital image procedures.

We have validated here that, as described when using the reporter in cultured cells, KFERQ-Dendra expressed by this mouse model is translocated and fully degraded in lysosomes and that its fluorescence when associated with the lysosomal membrane is independent of differences in lysosomal pH or in their proteolytic activity, since once unfolded for translocation the reporter is no longer fluorescent. There are however specific precautions that should be considered when using this mouse model outside the physiological context. Thus, although all changes in KFERQ-Dendra fluorescent puncta reported in this work were not due to changes at the transcriptional level, we recommend always measuring KFERQ-Dendra mRNA levels upon new manipulations or when examining new conditions. Similarly, although our studies in this mouse model confirm the correlation between lysosomal binding and uptake of substrates previously reported in vitro, it is advisable to confirm efficient lysosomal degradation of KFERQ-Dendra (as a read out of internalization) using inhibitors of lysosomal proteolysis. This could become important in pathological conditions such as Parkinson’s disease^10^, when substrate binding has been shown to be preserved but lysosomal translocation via CMA is halted by the pathogenic proteins.

We have validated here that association of this reporter with lysosomes is fully dependent on CMA activity, since the starvation-induced increase of this association is no longer observed in a CMA-incompetent genetic mouse model (Figs. 2g, h, 4b). In contrast, disruption of other autophagic pathways such as macroautophagy not only do not inhibit, but as reported before, upregulate fluorescent puncta formation, reflecting a compensatory increase in CMA (Supplementary Fig. 4a). Comparison of this mouse model with previously generated fluorescent mouse models for tracking macroautophagy (GFP-LC3 mice)^26^ or mitophagy (mito-QC^27^ and mito-Keima^28^ mice) also revealed specific differences in the cell intrinsic activity and response to stressors of these two autophagic pathways when compared to the CMA reporter mice. For example, basal macroautophagy in the kidney was shown to be mostly active in glomeruli^26^, whereas here we show that both basal and starvation-induced CMA in glomeruli is very low when compared to the high CMA activity observed in proximal tubules (Fig. 2a–c and Supplementary Fig. 3a, d). Interestingly, studies with the mito-QC mice, developed to track mitophagy in vivo demonstrated that proximal tubules also display higher mitophagic activity^27^. Studies with mito-Keima mice, another model for mitophagy tracking in vivo, also detected brain regional differences for mitophagy, but the mito-Keima images in the hippocampal region of mice show a rather even distribution of mitophagy events in this region^28^, whereas in this work we show maximal basal CMA activity in neurons in the CA2 and CA1 regions and lower activity in the dentate gyrus neurons (Fig. 3d). Future comparative analysis with these different autophagy reporter mice should help in gaining a better understanding of cell and tissue prevalence of each of these autophagy pathways under basal conditions and of their coordination in response to stress.

The favorable cytosol to lysosomal signal ratios found in most tissues in this transgenic mouse model makes photoswitching of Dendra from green to red (required in transfected cultured cells to track the lysosome-associated reporter against its cytosolic signal) unnecessary. However, the photoswtichable property of the Dendra protein used in this mouse model may become useful for tracking CMA before and after specific interventions in organotypic or primary cell cultures, as we have shown here for the response to oxidative stress in cultured primary astrocytes (Supplementary Fig. 4b).

We have presented examples of the versatility and possible uses of this mouse model for studies with whole animals in vivo, in fixed tissue sections, organotypic cultures and in primary cultures from cells isolated from these animals. Despite current limitations of intravital microscopy in the depth and level of resolution that can be attained in a live animal, even when movements due to breathing are minimized through immobilization and sedation, we showed that tracking dynamics of individual lysosomes active for CMA is possible in this model (Fig. 4a). Advanced imaging technologies may further expand the range of applications of this model and contribute to a more in-depth understanding of the properties and regulation of this selective form of autophagy. Considering the growing interest in CMA as a result of the tight connections between its malfunctioning and human diseases^10,29,30^, we anticipate that this mouse model will be of great value for those interested in analyzing spatial and temporal changes in CMA in the course of different diseases and for evaluating the efficiency of therapeutic interventions based on modulating CMA activity.

## Methods

### Animals and treatments

KFERQ-Dendra mice were generated by donor egg injection in wild-type FVB mice using the pRP.ExSi plasmid backbone with the insert coding for 11 amino acids including the KFERQ sequence of RNase A in frame with the sequence of Dendra 2 under the hybrid promoter CAGG as described in the text. Except where indicated, mice used for this study were FVB, males and females with ages ranging from 4 to 6 months of age. C57BL/6 KFERQ-Dendra mice were generated by back-crossing FVB KFERQ-Dendra mice with wild-type C57BL/6 mice for 8 generations. KFERQ-Dendra mice defective in CMA (KFERQ-Dendra-L2A^null^) were generated by breeding KFERQ-Dendra male mice with constitutive LAMP2A knock-out (L2A^null^)^19^ female mice to guarantee that all males were null for LAMP2A since this gene is located in the X chromosome. All animals were maintained in standard chow diet and where indicated, they were starved for 24 or 48 h by removing food but maintaining water ad libitum. For leupeptin treatment, mice were intraperitoneally (i.p.) injected with either PBS or leupeptin (30 mg/kg b.w.) (Sigma, L5793) by single injection at 12 h and 2 h before dissection and tissue collection. Chloroquine (50 mg/kg b.w.) was single injected by i.p. daily for two consecutive days and tissues collected 12 h after the second injection. For induction of mild oxidative stress, mice were i.p. single injected with paraquat (PQ) (20 mg/kg b.w.) for two consecutive days. For treatment with the CMA activator (CA; available in small amounts upon request from the investigators laboratory), mice were i.p. single injected with CA (15 mg/kg b.w.) (modified from^24^) or vehicle twice a day for 3 days and tissues collected 6 h after the last injection. Acetaminophen (Sigma, A7085; 400 mg/kg b.w.) was i.p. single injected and tissues collected after 16 h. Before tissue dissection, all animals were perfused with PBS. Allocation of animals in the vehicle or treatment group was done randomly. All animal studies and procedures complied with ethical regulations and were approved by the Institutional Animal Care and Use Committee at the Albert Einstein College of Medicine.

### Antibodies

Primary antibodies are from the following sources (dilution for immunofluorescence and clone indicated in brackets): rabbit anti Dendra2 (1/200 ANTIBODIES, abin361314), Rat anti LAMP1 (1/500 Hybridoma Bank, 1D4B), rat anti LAMP2 (dilution 1/500 Hybridoma Bank, GL2A7), rabbit anti LC3B (1/1000 MBL pm036), mouse anti β-actin (1/10000 Sigma, A4700), mouse anti Megalin (1/500 Santa Cruz, sc-515750), rat anti CD68 (1/200 Bio-Rad, mca1957), rabbit anti α-SMA (1/500 Abcam, ab124964), mouse anti MAP2 (1/1000, Sigma-Aldrich, M1406), rabbit anti GFAP (1/1000, DAKO, Z0334), rabbit anti IBA1 (1:1000, Wako, 19019741), rabbit anti HSC70 (1:2000, Assay Designs, adi-spa-757), Chicken anti Albumin (1:1000, Abcam, ab106582). All the secondary antibodies were purchased from Thermo Scientific. All antibodies used in this study were from commercial sources and were validated following the multiple dilution method and where available through the use of cell lines or tissues from animals knock-out for the antigen.

### Cell isolation and cultures

Cortical neurons were obtained from KFERQ-Dendra P0-P1 postnatal mice and neuronal cultures were prepared as follows^31^: brain cortices were dissected in cold PBS and enzymatically (0.25 % trypsin) and mechanically dissociated in neurobasal medium with 2% fetal bovine serum. After centrifugation, neurons were resuspended in Neurobasal Medium (ThermoFisher 10888022), supplemented with 2% B27-Supplement (Gibco-Invitrogen, 17504044), 1% Penicillin/Streptomycin and 1% GlutaMAX (Fisher, 35050-061). For immunostaining, cells (3 × 10^4^) were plated on 14 mm diameter coverslips pre-coated with poly-d-lysine, cultured for 7 days in vitro, and then fixed with 4% paraformaldehyde. To generate primary astrocyte cultures^32^ cells were isolated from mouse brain as above and then plated and maintained in DMEM, 10% Fetal Bovine Serum and 1% Penicillin/Streptomycin until confluence. Cultures were then split twice, and left under agitation for 2 h to remove the other contaminant cell types. Cells were then plated (20 × 10^3^ cells/well in 24-well plates) and seven days after different treatments were applied for 16 h. The following drugs/concentrations were used: 10 mM 3-Methyladenine (Sigma M9281), 20 nM VPS34IN (Selleckchem S7980), 5 μM CMA activator^24^, and 100 μM Paraquat (Sigma 856177). Photoswitching with a 405/20 nm LED array (Norlux) for 3 min was performed either immediately before or 12 h after treatment with Paraquat.

To isolate hepatocytes and Kupffer cells from mouse liver^33^, mice were perfused with HBSS-EGTA (0.5 mM) followed by warm collagenase (3.2 g/l in HBSS-CaCl_2_) and the cellular suspension was filtered through a 100 μm restrainer and hepatocytes collected by centrifugation (50*g* for 3 min at 4 °C). The Kupffer cells were recovered after subjecting the supernatant to centrifugation (1200*g* for 30 min at 4 °C) in a Percoll gradient and wased I RPMI media. Hepatocytes were washed 3 times in HBSS-CaCl_2_ and 3 times in adherence media M199. After plating for 3–4 h in M199 they were switched to M199 supplemented with Earles Salts, 100 nM Dexamethasone, 1% Penicillin/Streptomycin and 100 nM of Insulin. Purity of cell isolates was confirmed by immunoblot against albumin (hepatocytes) and CD-68 (Kupffer cells). NIH3T3 cells used in this study were obtained from the American Type Culture Collection and were validated by genomic PCR. All the cells lines were tested for mycoplasma contamination using DNA staining protocol with Hoechst 33258 dye.

### Organotypic brain cultures

Brain slices (200 µm thick) were obtained from 3 months old KFERQ-Dendra mouse brains subjected to coronal sectioning in vibratome and recovered in the ACSF solution (4 mM KCl, 26 mM NaHCO_3_, 1 mM CaCl_2,_ 5 mM MgCl_2_, 246 mM Sucrose, 10 mM D-Glucose) under continuous oxygenation. Slices were transferred to Millicell Cell culture insert (Millipore PICM01250) filled with culture medium (Neurobasal A with 2% B27, 2mM L-glutamine, and antibiotics-antimycotics). After 3 days culture sections were fixed for 12 h at 4 °C with 2% formaldehyde, 0.2% picric acid in PBS pH7.

### Isolation of subcellular fractions

Lysosomes with high activity for CMA were isolated from the indicated tissues after homogenization and differential centrifugation to recover a light mitochondrial-lysosomal enriched fraction that was then subjected to centrifugation in a discontinuous density metrizamide gradient by the modified method described previously^34^. Preparations with more than 10% broken lysosomes, measured by β-hexosaminidase latency, were discarded.

### In vitro CMA assay

Uptake and degradation of KFERQ-Dendra protein bound to the membrane of intact lysosomes isolated from livers and kidneys of KFERQ-Dendra transgenic mice were measured by immunoblot after incubation for 20 min at 37 °C in an isotonic media. Proteolytic activity was measured by incubating lysosomes with a [H^3^] radiolabeled pool of long-lived cytosolic proteins in the presence of 1% Triton X-100 to disrupt the lysosomal membrane as described before^35^. Proteolysis was calculated as the amount of added radiolabeled protein converted into free radiolabeled amino acids and small peptides by the end of the incubation.

### Immunostaining

Vibratome sections of the peripheral organs were fixed in 4% PFA for 30 min and permeabilized for 12 h with 0.5% TritonX-100 in PBS. Slices were incubated with 2% Goat serum, 2% donkey serum, 2% BSA in PBS blocking buffer for 12 h at 4 °C or 1 h at room temperature (RT). For brain, slices were fixed in aqueous buffered zinc formalin fixative (Anatech LTD) for 12 h at RT, washed in PBS and blocked for 1 h. After blocking, slices were incubated for 12 h at 4 °C with the indicated primary antibody followed by 1 h incubation with the corresponding secondary antibody. Slices were mounted in DAPI-Fluoromount-G (SouthernBiotech) to highlight the cell nucleus. For primary cells, cells grown on coverslips were fixed and permeabilized in 0.1%Triton, 4% Bovine Serum Albumin in PBS for 1 h at 25 °C and then incubated with the primary antibody diluted in blocking buffer for 12 h at 4 °C. After extensive washing in PBS/0.1%Triton, coverslips were incubated for 1 h at 25 °C with the corresponding secondary antibody, then incubated with DAPI and mounted in ProLong Diamond Antifade Mountant (Invitrogen). In the case of the brain slices from organotypic cultures, the slices were permeabilized in 0. 5% Triton-PBS for 12 h at 4 °C, blocked in 20% BSA solution for 4 h at 25 °C and subsequently incubated with the primary and secondary antibodies as above but extending the incubation with the secondary antibody to 3 h at 25 °C. The slices were incubated with DAPI and mounted in ProLong Diamond Antifade Mountant (Invitrogen). Isolated lysosomes were imaged after seeding on printed coverslips and following the same procedures as for cultured cells.

### Green Dendra fluorescence imaging

Tissues were either processed using the vibratome method described above or the OCT embedding method as following: Organs of interest were fixed for 12 h at 4 °C in picric acid fixation buffer (2% formaldehyde, 0.2% picric acid in PBS, pH7.0) and then washed with 70% ethanol, followed by two washes in PBS. Tissues were immersed in 30% sucrose and then embedded in the OCT for sectioning in a cryostat (Leica CM3050 S). After air-drying for 30 min, sections were stored at −20 °C until use. Slices were mounted in DAPI-Fluoromount-G to highlight the cell nucleus. Most of the direct fluorescence images were obtained through the vibratome method unless otherwise labeled the OCT method in the figure legend. Images were acquired in *x*–*y*–*z* planes with an Axiovert 200 fluorescence microscope (Carl Zeiss Ltd) with a ×63 objective and 1.4 numerical aperture, mounted with an ApoTome.2 slider or with a Confocal microscope (TCS SP5; Leica) using an HCX Plan Apo CS ×63.0 1.40 NA oil objective an the Leica Application Suite X (LAS X).

### Two-photon microscopy Imaging

Two-photon microscopy imaging was performed using an Olympus FV1000 multiphoton microscope with a ×25 1.05 NA water immersion objective. ﻿Briefly, a Coherent Chameleon Vision II laser was tuned to 880 nm for excitation of GFP. Collagen was visualized by second harmonic generation. 3D images were reconstructed with Imaris 8 3.0 (Bitplane).

### Real-time intravital imaging

Mice were anesthetized with isoflurane and liver tissue was exposed by performing a small incision in the abdominal cavity^36^. Imaging was performed using an Olympus FV1000 multiphoton microscope with a ×25 1.05 NA water immersion objective. A Coherent Chameleon Vision II laser was tuned to 880 nm for excitation of GFP. Where indicated, mice were injected i.v. with fluorescent dextran (TRITC) to highlight endo/lysosomal compartments.

### Tissue FISH and imaging

Frozen liver sections were fixed with 4% (vol/vol) paraformaldehyde in 1x PBS for 15 min and then incubated in cold 70% ethanol for a minimum of 2 h before proceeding to hybridization^37^. A treatment with proteinase K (Ambion) in 2× SSC buffer (Roche) was included to improve signal-to-noise ratio. After incubation with 15% (vol/vol) formamide and 2× SSC in RNase-free water, tissue sections were incubated overnight at 37 °C with 50 nM Dendra probes (Stellaris RNA FISH Probes from LGC Biosearch Technologies) labeled with Quasar 670 fluorescent dye and complementary to the coding sequence of dendra gene, in Hybridization Buffer (15% (vol/vol) formamide, 1 mg/mL *Escherichia col*i tRNA, 10% dextran sulfate, 0.2 mg/mL BSA, 2× SSC, 2 mM Vanadyl Ribonucleoside Complex, in RNase-free water). The following day, tissue sections were washed in 15% formamide and 2x SSC in RNase-free water. DNA was counterstained with DAPI (50 ng/ml, Sigma-Aldrich) during the washes. ProLong Diamond Antifade Reagent (Life Technologies) was used for mounting the slides. Images were taken using an upright, wide-field Olympus BX- 63 Microscope equipped with a SuperApochromatic objective lens (60×/1.35 N.A.). FISH probe sequences are available in supplementary Table 1.

### High-content microscopy

Mouse fibroblasts transduced with lentivirus particles carrying KFERQ-PS-Dendra2 were plated in glass-bottom 96-well plates and photoactivated with a 405 nm light emitting diode (LED: Norlux) for 3 min with the intensity of 3.5 mA (current constant). After 16 h, cells were fixed with 4% paraformaldehyde and images were captured with a high-content microscope (Operetta system, Perkin Elmer) and quantification was performed with the manufacturer’s software in a minimum of 1,200 cells (approx. 9 fields).

### Image analysis

Investigators were blinded to the treatment during data collection and analysis and unblinding was done when the analysis was completed for plotting. Quantification was performed on non-thresholded original orthogonal Z-projections generated in ImageJ software for a minimum of 25 cells per slide. The particle number were quantified with ImageJ 1.5j8 (NIH) with size (pixel^2^) settings from 0.1 to 10 and circularity from 0 to 1. For liver, kidney, WAT and BAT tissues, Dendra-positive and LAMP1-positive lysosomes with intensity above cytoplasmic background and size (pixel^2^) from 2 to 10 were quantified. Representative images were processed in Adobe Photoshop CS3 software (Adobe Systems Inc.) where brightness/contrast was adjusted equally on images being compared. FISH images were quantified with ImageJ 1.5j8 (NIH). A threshold was applied on each cell (Yen white) and particles with size (pixel^2^) settings between 6 and 100 (that corresponds to 0.645–1.07 μm) and circularity from 0 to 1 were quantified. A total of 40–50 cells per layer were analyzed.

### H&E staining

Livers from acetaminophen or PBS treated mice were fixed in 10% neutral buffered formalin and stained with H&E using standard procedures.

### Electrophoresis and immunoblotting

Mice tissues were homogenized in a Teflon/glass motorized homogenized by 10 strokes and protein in the homogenate was extracted with RIPA buffer (50 mM TrisHCl pH7.4, 150 mM NaCl, 2 mM EDTA, 1% TritonX-100, 0.1% SDS) supplemented with protease inhibitors. The homogenates were centrifuged at 500 *g* at 4 °C for 10 min (for white adipose tissue the homogenates were centrifuged at 12,000 *g* for 15 min). Samples were subjected to SDS-PAGE, transferred to nitrocellulose membrane, blocked with low-fat milk and incubated with primary antibody for 12 h^38^. The proteins of interest were visualized by using peroxidase-conjugated secondary antibodies and chemiluminescent reagent (PerkinElmer) in LAS-3000 Imaging System (Fujifilm). Densitometric quantification was performed on unsaturated images using ImageJ software (NIH). Images of uncropped and unprocessed scans of all immunoblots in main and supplementary figures are included as Supplementary Fig. 6.

### Quantitative PCR

mRNA was isolated from different tissues (10 mg) using RNeasy Plus Mini Kit (QIAGEN). Transcripts were reverse transcribed according to manufacturer protocols (Invitrogen), and qPCR was performed using SYBR Green (Applied Biosystems). The following primers were used: mouse β-actin, F-5′-AAGGACTCCTATAGTGGGTGACGA-3′, R-5′-ATCTTCT CCATGTCGTCCCAGTTG; mouse TBP, F-5′-GTTGGGCTTCCCAGCTAAGT-3′, R-5′-CACAAGGCCT TCCAGCCTTA-3′; mouse HPRT, F-5′-ACAGGCCAGACTTTGTTGGA-3′, R-5′-ACTGGCAACATCAAC AGGACT-3′; mouse 18 S rRNA, F-5′-GTAACCCGTTGAACCCCATT-3′, R-5′- CCATCCAATCGGTA GTAGCG-3′; Dendra2, F-5′-AAGGGCATCTGCACCATCCG-3′, R-5′-CGTGCTCGTACAGCT TCACCTTG-3′.

### Statistical analysis

All numerical results are reported as mean ± s.e.m. and represent data from a minimum of three independent experiments unless otherwise stated. We determined the statistical significance in instances of single comparisons by the two-tailed unpaired Student’s *t* test of the means with Prism software 8.3.0 (Graph Pad Software Inc). One-way ANOVA test followed by Tukey’s multiple comparisons test (when compare the mean of each conditon with every other condition) or Dunnett’s multiple comparisons test (when compare the mean of each condition with control condition) was used for comparisons with more than two groups and two-way ANOVA test followed by Sidak’s multiple comparisons test to test differences in means across different tissues and conditions. All the statistical tests are two-sided. All the one-way and two-way ANOVA tests are corrected for multiple comparisons. Statistical analysis was performed in all the assays, and significant differences are noted in the graphical representations. No data were excluded in any of the experiments. All the tests are null hypothesis testing. The test statistic (*F*, *t*, *R*), effect size (calculated from Cohen’s d), confidence interval and degrees of freedom are indicated in Supplementary Data 1. Assumption for data sampled from populations that follow Gaussian distributions was tested using the method Kolmogorov and Smirnov. For the studies in cell lines in culture and in vitro assays we determine number of experimental repetitions to account for technical variability and changes in culture conditions. The number of animals used for experiment was calculated through power analysis based in previous results on changes in CMA under different conditions in liver and kidney. For the studies of isolation of organelles from animals, the number of specimens used was determined based on the average values of enrichment and recovery for the specific fraction using endogenous markers for each compartment from our previous studies.

### Reporting summary

Further information on research design is available in the Nature Research Reporting Summary linked to this article.

## Supplementary information

Supplementary Information

Description of Additional Supplementary Files

Supplementary Data 1

Supplementary Movie 1

Supplementary Movie 2

Supplementary Movie 3

Supplementary Movie 4

Supplementary Movie 5

Supplementary Movie 6

Reporting Summary

## Data Availability

There are not restrictions on data availability in this manuscript. All the information is included in the manuscript. Figs. 1, 2, 4 and 5 and supplementary figs. 1–5 have associated raw data that is provided as an excel worksheet organized by figures in Source Data.

## Source data

Source Data
