# Supplementary material for: Monitoring spatiotemporal changes in chaperone-mediated autophagy in vivo
Source: Nat Commun. 2020 Jan 31;11:645. doi: 10.1038/s41467-019-14164-4 (PMC6994528; doi:10.1038/s41467-019-14164-4)
Supplement: Supplementary file 1 — Supplementary Information [file 41467_2019_14164_MOESM1_ESM.pdf]

Supplementary Information

Monitoring spatiotemporal changes in chaperone-mediated autophagy in vivo

Dong et al.

Supplementary Figures 1-6

Supplementary Table 1

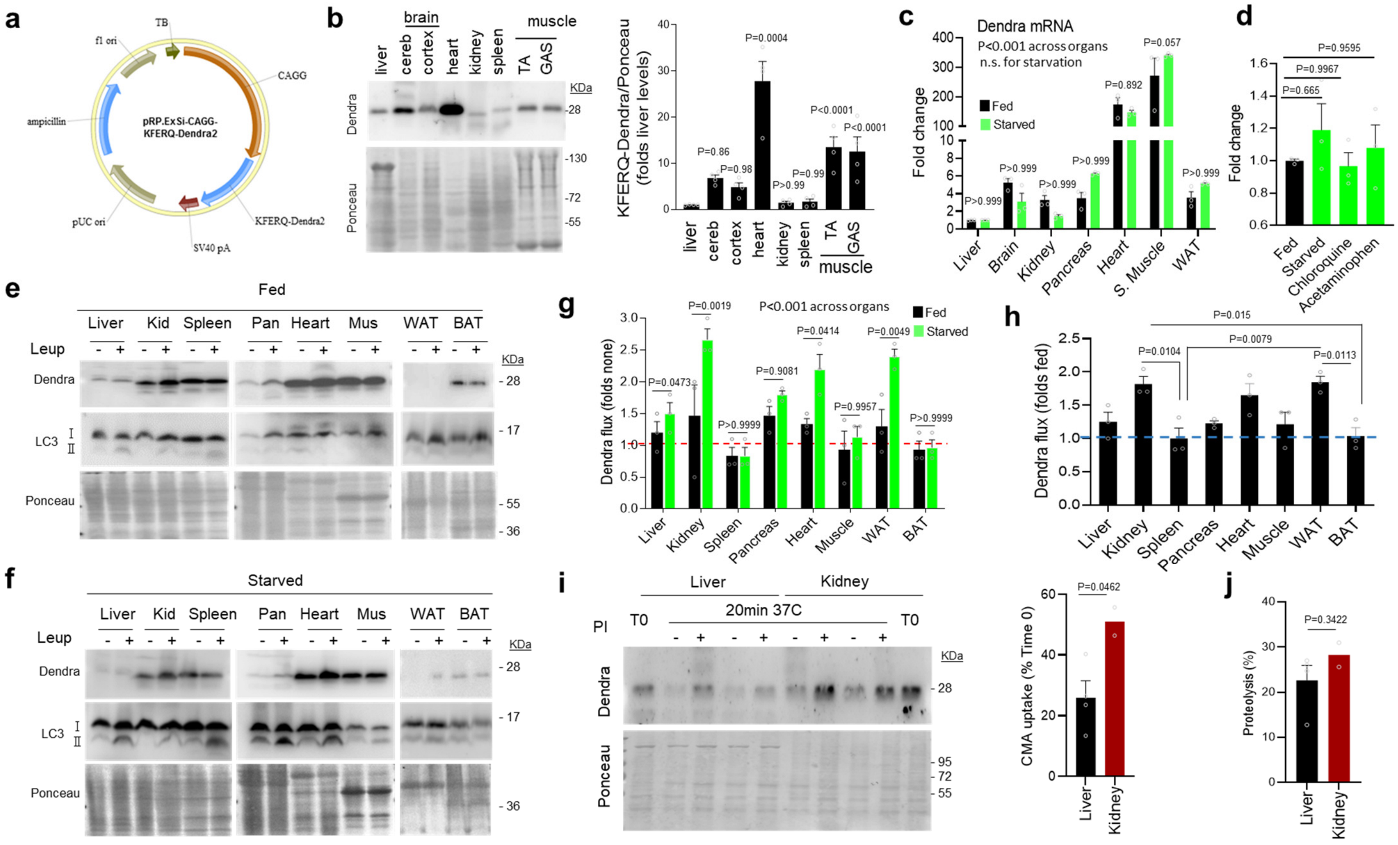

Supplementary Figure 1 Characterization of the KFERQ-Dendra mouse model. a. Scheme of the vector used for generation of the KFERQ-Dendra transgenic mouse model. **b.** Representative immunoblot for Dendra of the indicated tissues from KFERQ-Dendra mice and Dendra levels relative to those in liver. Similar results were obtained in 4 independent experiments. **c,d.** Quantitative PCR for Dendra in the indicated tissues relative to the average of four different housekeeping genes in fed and starved conditions, n=3 mice (**c**) or upon the indicated treatments, n=4 mice (**d**). Data points are values from different mice. **e,f.** Immunoblot for Dendra in tissues from fed (**e**) or starved (**f**) KFERQ-Dendra mice injected with PBS (**-**) or leupeptin (+)12 h and 2 h before tissue collection. LC3 is shown as positive control for leupeptin and Ponceau as loading control. **g,h.** Dendra protein degradation in the indicated tissues calculated as the difference between protein levels in presence and absence of leupeptin from the quantification of blots as the ones in e and f. Values are expressed relative to levels in non-injected mice (**g**) or as effect of 24h starvation on Dendra degradation in the same tissues (**h**), n=3 mice for both fed and starved conditions. **i,j.** Comparison of CMA (**i**) and lysosomal proteolytic activity (**j**) in lysosomes isolated from livers and kidneys of KFERQ-Dendra mice. (**i**) Immunoblot for Dendra protein after incubation in presence (+) or not (-) of protease inhibitors (PI) (**i,** left) and quantification of Dendra uptake by lysosomes expressed as percentage of time 0, n=4 mice for liver, n=2 mice for kidney (**i,** right). (**j**) Proteolysis of radiolabeled proteins by the same liver and kidney lysosomes upon disruption of their membrane with Triton X-100, n= 2 mice for kidney and 4 mice for liver. All the results are mean + s.e.m. T-test (**i, j**), One-way ANOVA followed by Tukey's multiple comparisons test (**b, d, h**) and Two-way ANOVA test followed by Sidak's multiple comparisons test (**c, g**) were used. Source data are provided as a Source Data file.

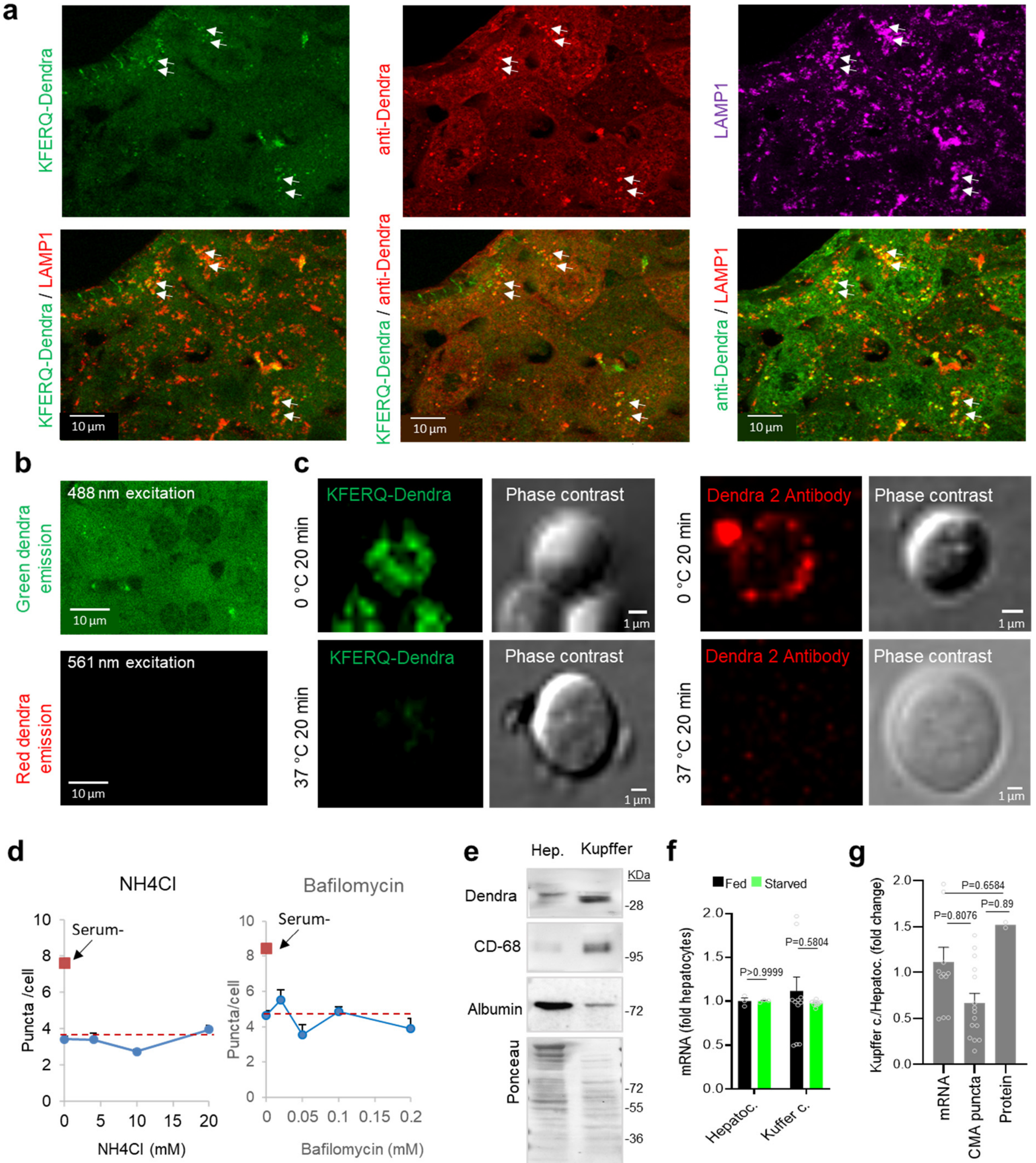

Supplementary Figure 2. Lysosome-associated KFERQ-Dendra. a. Green fluorescence for KFERQ-Dendra and immunostaining using Dendra and LAMP1 antibodies in livers from 24h starved KFERQ-Dendra mice. Bottom: merged images. White arrows indicate GFP Dendra/LAMP1/Dendra antibody (anti-Dendra) positive structures. Similar results were obtained in 4 independent experiments. **b.** Images of unstained livers from KFERQ-Dendra mice using a 488 nm laser. Both green (top) and red (bottom) channels are shown. Similar results were obtained in 3 independent experiments. **c.** Images of lysosomes isolated from livers of KFERQ-Dendra mice that were glass-spotted after incubation at 0 and 37°C. Left: direct fluorescence. Right: fluorescence upon incubation with the anti-dendra antibody and a far-red-conjugated

secondary antibody. Right shows phase contrast images. Similar results were obtained in 3 independent experiments. **d.** Mouse fibroblasts stably expressing KFERQ-Dendra were photo-switched and incubated for 16h with increasing concentrations of NH_4Cl (left) or bafilomycin (right). CMA was calculated as the average number of red fluorescent puncta per cell. Average of puncta in cells incubated in absence of serum for the same time is shown as positive control. Dotted line marks puncta in cells incubated without treatments. Values are average of 3 different experiments with approximately 1,200 cells counted per condition. **e-g.** Hepatocytes and Kupffer cells isolated from KFERQ-Dendra mice were subjected to immunoblot for Dendra, Albumin (hepatocyte marker) and CD68 (Kupffer cell marker) (**e**), quantitative PCR for Dendra relative to the average of four housekeeping genes in the same cells isolated from fed or 24h starved mice (**f**). n=3 independent experiments. Values in g show the ratio of Dendra mRNA (from S2f), Dendra-positive fluorescent puncta (from Fig. 1f, n=3 independent experiments) and Dendra protein (from S2e) in Kupffer cells relative to hepatocytes. Data are presented as mean values + s.e.m. One-way ANOVA test followed by Tukey's multiple comparisons test was used, but no significant differences were detected in **f** or **g**. Source data are provided as a Source Data file.

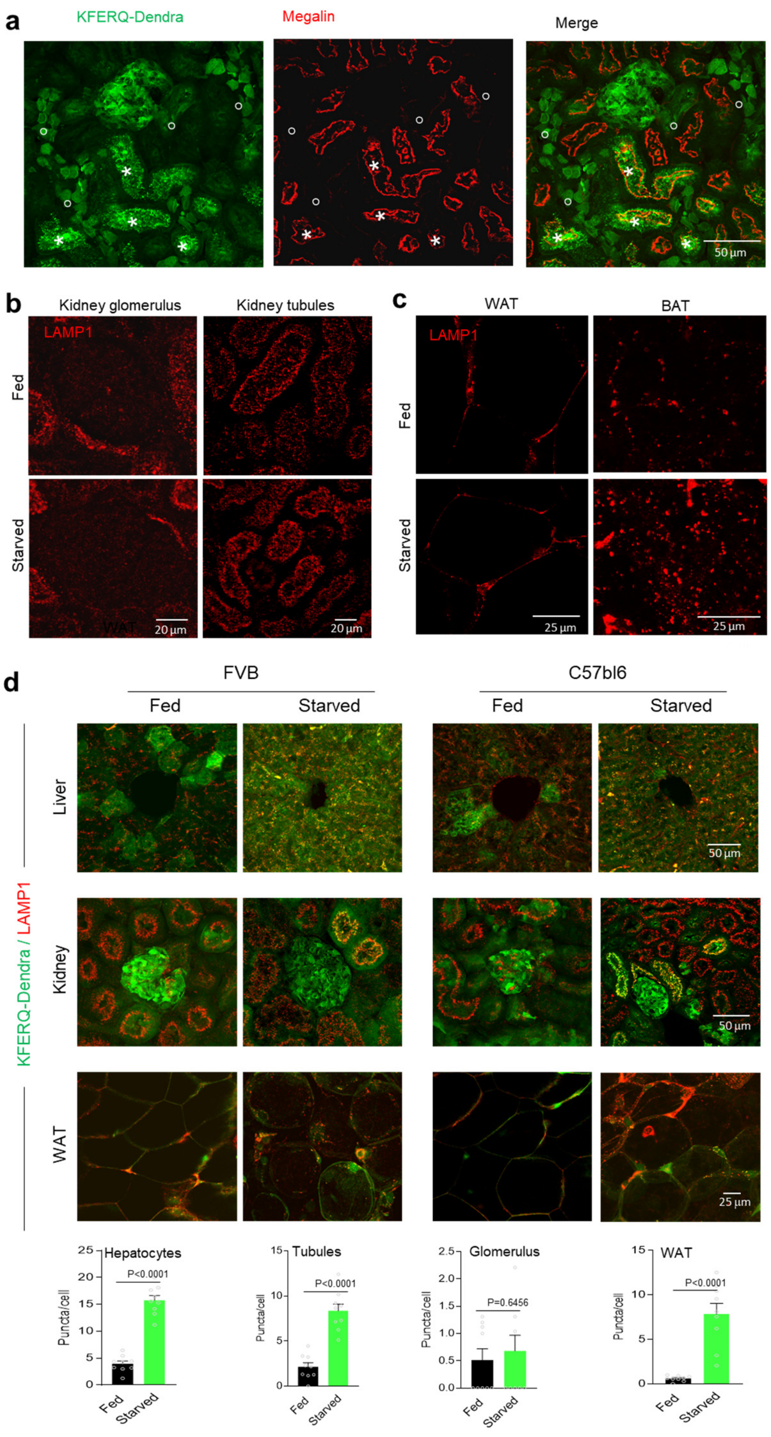

Supplementary Figure 3. CMA activity in kidney and adipose tissue. a. Green fluorescence for KFERQ-Dendra in kidney sections from 24h starved KFERQ-Dendra mice immunostained for Megalin. Single and merged channels are shown. Start denotes proximal tubules (Megalin-positive) and circle distal tubules (Megalin-negative). Similar results were obtained in 5 independent experiments. **b,c.** Sections of kidney (**b**), white and brown adipose tissue (**c**) from fed or 24h starved KFERQ-Dendra mice immunostained for LAMP1. Similar results were obtained in 5 independent experiments. **d.** Green fluorescence for KFERQ-Dendra and immunostaining

using LAMP1 antibody in liver, kidney and WAT from fed or 24h starved KFERQ-Dendra mice in an FVB background (left) or C57BL/6 (right) background. Merged images are shown. Bottom: Quantification of the Dendra-positive puncta in hepatocytes, kidney tubules, glomerulus and WAT of fed and starved C57BL/6 KFERQ-Dendra mice. Similar results were obtained in 3 independent experiments. Data point are average values from different sections (n =9 sections for each tissue per condition). Data are presented as mean values + s.e.m. Two tailed unpaired t-test was used. Source data are provided as a Source Data file.

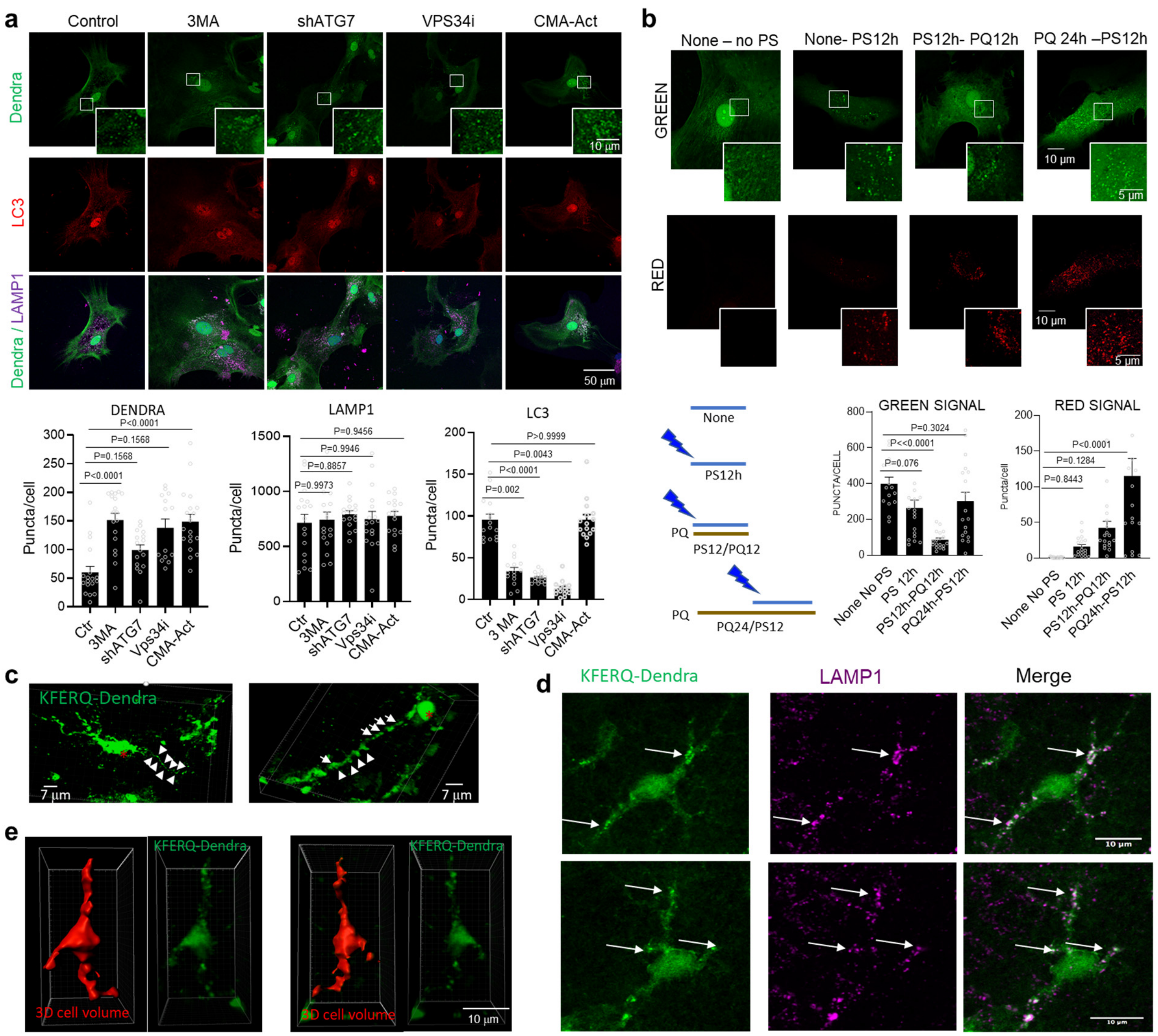

Supplementary Figure 4 CMA activity in KFERQ-Dendra mice brains. a. KFERQ-Dendra fluorescence and immunofluorescence staining for LC3 and LAMP1 in primary cultured astrocytes isolated from KFERQ-Dendra mice subjected to the indicated treatments: 3-methyladenine (3MA), shRNA against ATG7 (shATG7) or inhibitor of Vacuolar Protein Sorting 34 Inhibitor (Vps34i) to decrease macroautophagy and retinoid-derived small molecule activator of CMA (CMA-Act) to activate CMA. Top: representative images of single and merged channels. Insets show higher magnification. Bottom: Quantification of average number of Dendra fluorescent puncta (right), LAMP1 (middle) and LC3 (right) per cell. >25 cells were counted from 3 independent experiments. **b.** Primary cultured astrocytes untreated (None) or exposed to 100µM paraquat (PQ) for 12 or 24h (PQ12, PQ24) were photo-switched (PS) at time 0h (PS12h-PQ12h) or 12h after addition of PQ (PQ24h-PS12h). Images in the green (top), and red (bottom) channel are shown at the top. Insets show higher magnification regions. Bottom: Experimental scheme (left) and quantification of green (middle) or red (right) KFERQ-Dendra positive puncta per cell. Similar results were obtained in 3 independent experiments. Data point are average values from 3 separate wells in 3 independent experiments for each condition (n =18) **c.** 3D reconstruction of two-photon images of brain from KFERQ-Dendra mice to show full projection of brain cells

(microglia – left and neuron – right). Arrows: Dendra-positive puncta. Similar results were obtained in 3 independent experiments. **d.** KFERQ-Dendra fluorescence and immunofluorescence (for LAMP1) of brains from KFERQ-Dendra mice to show examples of brain cells displaying KFERQ-Dendra positive lysosomes (arrows). Similar results were obtained in 4 independent experiments. **e.** 3D reconstruction of cell volume (red) and Dendra puncta using two-photon images of a cortical neuron from KFERQ-Dendra mice to show that puncta can be detected in the cell projections. Two different orientations are shown (see also Movie 2). Similar results were obtained in 4 independent experiments. Data are presented as mean values + s.e.m. One-way ANOVA test followed by Tukey's multiple comparisons tests were used.Source data are provided as a Source Data file.

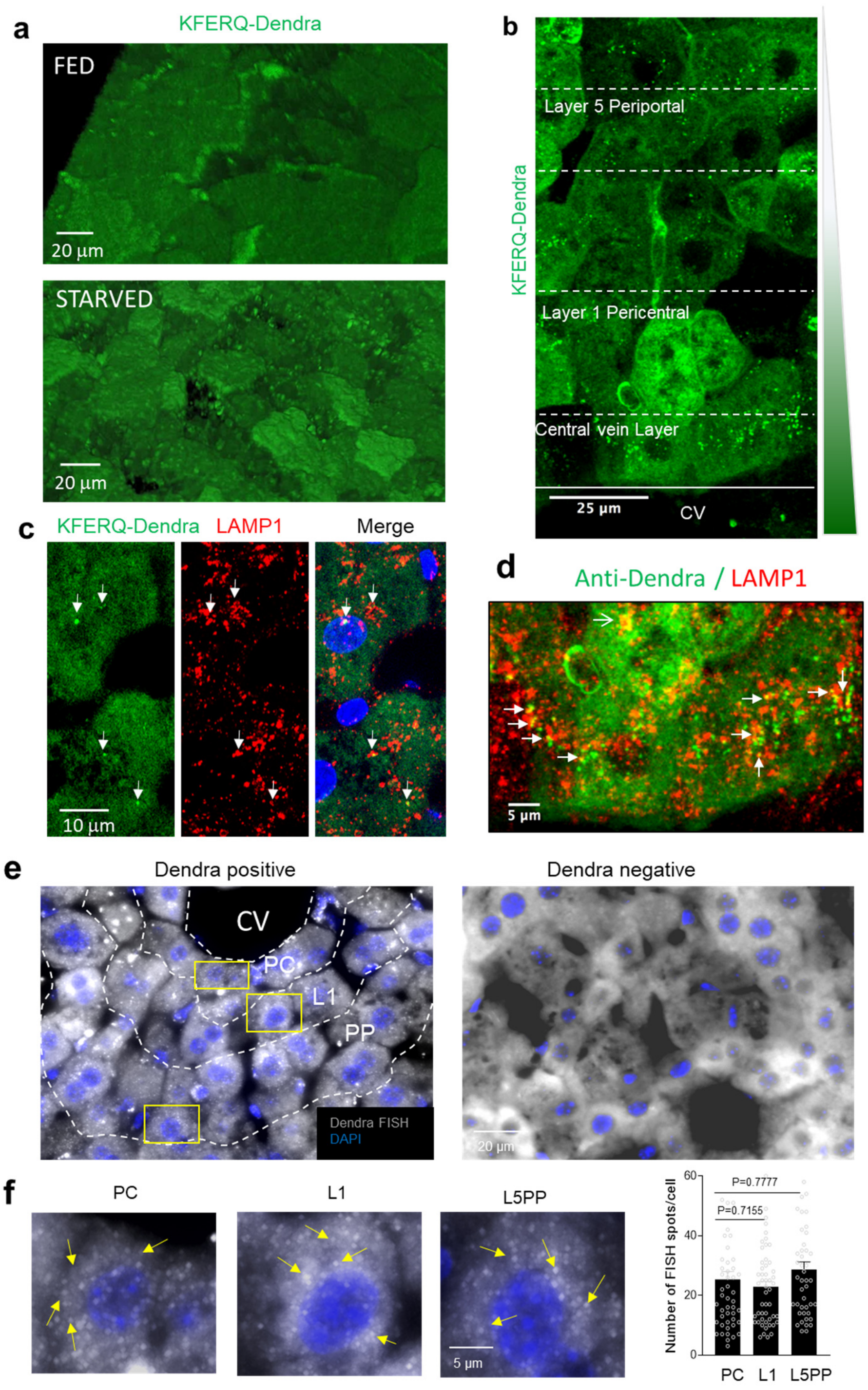

Supplementary Figure 5 High-resolution imaging of livers of KFERQ-Dendra mice a. 3D reconstruction of two-photon images of livers from fed and 24h starved KFERQ-Dendra mice. **b.** Higher magnification of zonation of livers from KFERQ-Dendra mice. **c.** KFERQ-Dendra fluorescence and immunofluorescence for LAMP1 in periportal regions of livers from fed KFERQ-Dendra mice. **d.** Immunostaining for Dendra (Dendra Ab) and LAMP1 in the periportal region. Arrows in b and c: examples of LAMP1 puncta positive for Dendra fluorescence. Similar results were obtained for a-d in 3 independent experiments. **e.** Fluorescence In Situ Hybridization (FISH)

with Dendra probes in liver from both Dendra positive (left) and Dendra negative (control) mice (right). smFISH spot signals were dilated by one pixel for visualization. **f.** Quantification of FISH spots/cell in different liver regions (CV: central vein layer; L1 PC: layer 1 pericentral, L5 PP: layer 5 periportal. Left: Representative images of the yellow square indicated regions in **e**. Yellow arrow indicates examples of single molecules of KFERQ-Dendra mRNA. Similar results were obtained in 5 independent experiments. Data points indicate average values of each section from 5 independent experiments (n=49, n=51, n=45 sections separately for PC, L1 and L5PP). Data are presented as mean values + s.e.m. One-way ANOVA test followed by Tukey's multiple comparisons were used.Source data are provided as a Source Data file.

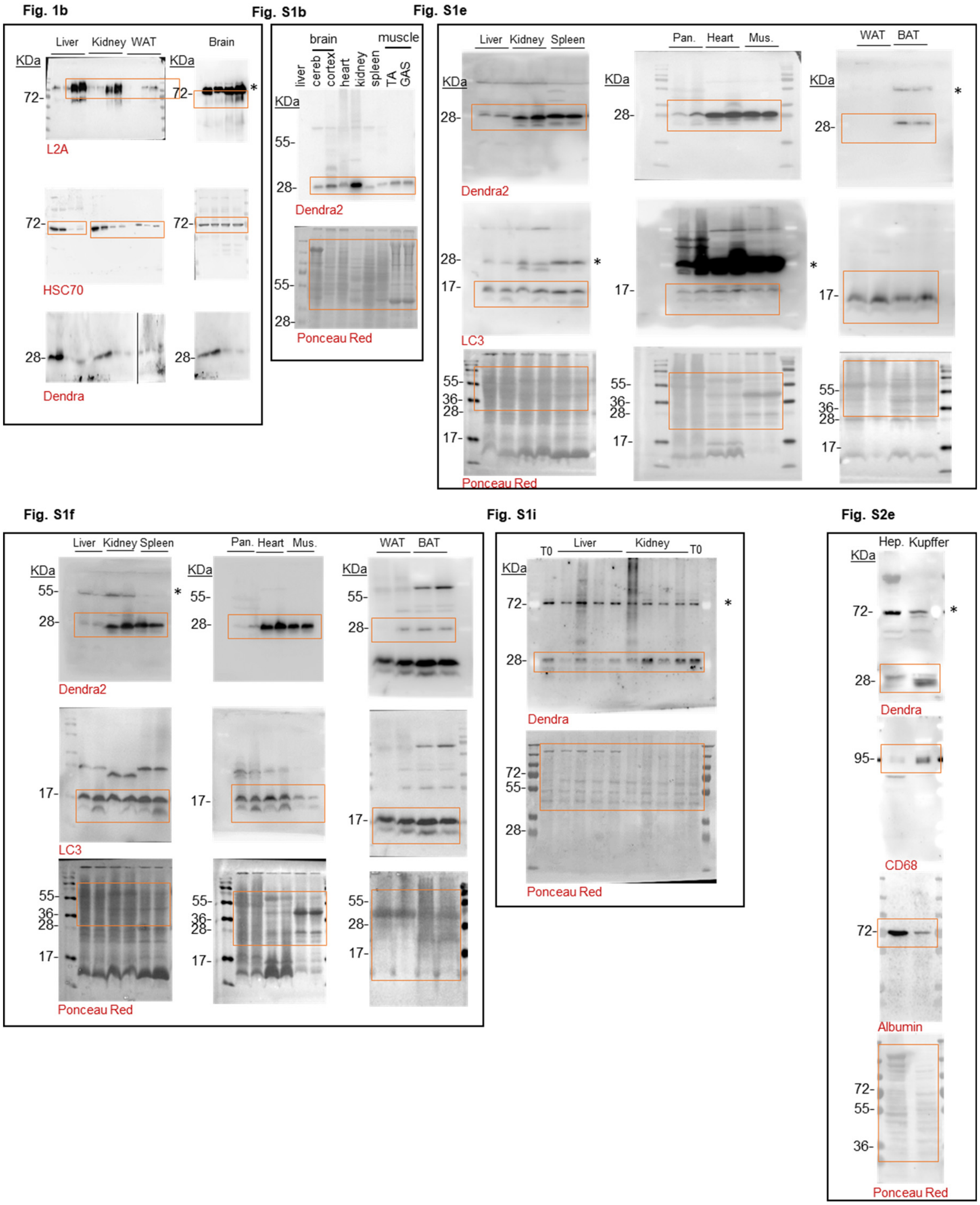

Supplementary Fig. 6. Uncropped blots: Full blots of sections shown in Fig. 1 and Supplementary Figs. 1, 2. Red box: area of the blot shown in the figure. * denotes non-related protein. Molecular weight of the markers are marked in the blots.

Supplementary Table 1: Dendra probes for FISH.

Dendra2 probes	
Dendra2_1	taattcccggggtgttca
Dendra2_2	catgtcctccttgatcag
Dendra2_3	tccatgtgcaccttcacg
Dendra2_4	cgatcacgaaggcgtggc
Dendra2_5	tccttcacggtcaggttg
Dendra2_6	gtagctgaagggcagggg
Dendra2_7	acggcggtggtcaggatg
Dendra2_8	aacacccggttgccgtag
Dendra2_9	tgtcctcggggtacttgg
Dendra2_10	ctgcttgaagtagtcggg
Dendra2_11	ctgtagccctcggggaag
Dendra2_12	ctcgaaggtcatggtgcg
Dendra2_13	ctgcggatggtgcagatg
Dendra2_14	gaagaagcagtcgccctc
Dendra2_15	cccttgaagcgcacgttc
Dendra2_16	cgttggggggaagttgg
Dendra2_17	ttcagggtcttcttctgc
Dendra2_18	acgtgcagcttctcggtg
Dendra2_19	cagggccatgttgatgtt
Dendra2_20	tcgcacaggtagtggccg
Dendra2_21	ccttgtaggtggtcttga
Dendra2_22	cagctgcaccaccttctt
Dendra2_23	tccacgaagtgggcgtcg
Dendra2_24	ttgcccaggatctcgatg
Dendra2_25	tcaccttgttgtagtcgc
Dendra2_26	cacggcgtgctcgtacag
