# Supplementary material for: Monitoring spatiotemporal changes in chaperone-mediated autophagy in vivo
Source: Nat Commun. 2020 Jan 31;11:645. doi: 10.1038/s41467-019-14164-4 (PMC6994528; doi:10.1038/s41467-019-14164-4)
Supplement: Supplementary file 2 — Description of Additional Supplementary Files [file 41467_2019_14164_MOESM2_ESM.pdf]

Description of Additional Supplementary files

File Name: Supplementary Movie 1

Description: 3D reconstruction of two-photon image of a single neuron from KFERQDendra mice brains.

File Name: Supplementary Movie 2

Description: 3D reconstruction of two-photon image of a single cortical neuron showing cell volume in red and green fluorescence puncta from KFERQ-Dendra mice brains.

File Name: Supplementary Movie 3

Description : 3D reconstruction of two-photon image of a single glial cell from KFERQ-Dendra mice brains.

File Name: Supplementary Movie 4

Description: 3D reconstruction of two-photon image from hippocampus region of KFERQ-Dendra mice brains.

File Name: Supplementary Movie 5

Description: 3D reconstruction of two-photon images of kidneys of fed (0”-24”) or 24h starved (25”-41”) KFERQ-Dendra mice. Green: KFERQ-dendra; Blue: Collagen Second harmonic generation.

File Name: Supplementary Movie 6

Description: 3D reconstruction of two-photon images of livers of fed (0”-22”) or 24h starved (23”-45”) KFERQ-Dendra mice. Green: KFERQ-dendra; Blue: Collagen Second harmonic generation.

File Name: Supplementary Data 1

Description: Includes all the statistic tests (F, T, R) with confidence intervals, effect sizes, degrees of freedom for all Main and Supplementary Figures.
