# Supplementary material for: Monitoring spatiotemporal changes in chaperone-mediated autophagy in vivo
Source: Nat Commun. 2020 Jan 31;11:645. doi: 10.1038/s41467-019-14164-4 (PMC6994528; doi:10.1038/s41467-019-14164-4)
Supplement: Supplementary file 10 — Reporting Summary [file 41467_2019_14164_MOESM10_ESM.pdf]

nature research

Corresponding author(s): Ana Maria Cuervo
Jose Javier Bravo-Cordero
Esperanza Arias

Reporting Summary

Nature Research wishes to improve the reproducibility of the work that we publish. This form provides structure for consistency and transparency in reporting. For further information on Nature Research policies, see Authors & Referees and the Editorial Policy Checklist.

Statistical parameters

When statistical analyses are reported, confirm that the following items are present in the relevant location (e.g. figure legend, table legend, main text, or Methods section).

n/a	Confirmed	
☐	☒	The exact sample size (n) for each experimental group/condition, given as a discrete number and unit of measurement
☐	☒	An indication of whether measurements were taken from distinct samples or whether the same sample was measured repeatedly
☐	☒	The statistical test(s) used AND whether they are one- or two-sided<br>*Only common tests should be described solely by name; describe more complex techniques in the Methods section.*
☐	☒	A description of all covariates tested
☐	☒	A description of any assumptions or corrections, such as tests of normality and adjustment for multiple comparisons
☐	☒	A full description of the statistics including central tendency (e.g. means) or other basic estimates (e.g. regression coefficient) AND variation (e.g. standard deviation) or associated estimates of uncertainty (e.g. confidence intervals)
☐	☒	For null hypothesis testing, the test statistic (e.g. F, t, r) with confidence intervals, effect sizes, degrees of freedom and P value noted<br>*Give P values as exact values whenever suitable.*
☐	☒	For Bayesian analysis, information on the choice of priors and Markov chain Monte Carlo settings
☒	☐	For hierarchical and complex designs, identification of the appropriate level for tests and full reporting of outcomes
☐	☒	Estimates of effect sizes (e.g. Cohen's d, Pearson's r), indicating how they were calculated
☐	☒	Clearly defined error bars<br>*State explicitly what error bars represent (e.g. SD, SE, CI)*

Our web collection on statistics for biologists may be useful.

Software and code

Policy information about availability of computer code

Data collection	Adobe Photoshop CS3 software (Adobe Systems Inc.)
Data analysis	ImageJ 1.5j8 (NIH) ; Imaris 8.3.0 (Bitplane); Leica Application Suite X (LAS X); Prism software 7.04 (Graph Pad Software Inc)

For manuscripts utilizing custom algorithms or software that are central to the research but not yet described in published literature, software must be made available to editors/reviewers upon request. We strongly encourage code deposition in a community repository (e.g. GitHub). See the Nature Research guidelines for submitting code & software for further information.

Data

Policy information about availability of data

All manuscripts must include a data availability statement. This statement should provide the following information, where applicable:

- Accession codes, unique identifiers, or web links for publicly available datasets
- A list of figures that have associated raw data
- A description of any restrictions on data availability

Page 27 reads: There are not restrictions on data availability in this manuscript. All the information is included in the manuscript. Figures 1, 2, 4 and 5 and supplementary figures 1-5 have associated raw data that is provided as an excel worksheet organized by figures in Source Data.

Field-specific reporting

Please select the best fit for your research. If you are not sure, read the appropriate sections before making your selection.

☒ Life sciences ☐ Behavioural & social sciences ☐ Ecological, evolutionary & environmental sciences

For a reference copy of the document with all sections, see nature.com/authors/policies/ReportingSummary-flat.pdf

Life sciences study design

All studies must disclose on these points even when the disclosure is negative.

Sample size	Page 26 indicates: For the studies in cells lines in culture and in vitro assays we determine number of experimental repetitions to account for technical variability and changes in culture conditions.The number of animals used for experiment was calculated through power analysis based in previous results on changes in CMA under different conditions in liver and kidney. For the studies of isolation of organelles from animals, the number of specimens used was determined based on the average values of enrichment and recovery for the specific fraction using endogenous markers for each compartment from our previous studies.
Data exclusions	Page 26 indicates: No data was excluded in any of the experiments.
Replication	Number of replicates have been stated in each of the panels for each figure.
Randomization	Page 19 states: Allocation of animals in the vehicle or treatment group was done randomly
Blinding	Page 24 states: Investigators were blinded to the treatment during data collection and analysis and unblinding was done when the analysis was completed for plotting.

Reporting for specific materials, systems and methods

Materials & experimental systems

n/a	Involved in the study
☐	☒ Unique biological materials
☐	☒ Antibodies
☐	☒ Eukaryotic cell lines
☒	☐ Palaeontology
☐	☒ Animals and other organisms
☒	☐ Human research participants

Methods

n/a	Involved in the study
☒	☐ ChIP-seq
☒	☐ Flow cytometry
☒	☐ MRI-based neuroimaging

Unique biological materials

Policy information about availability of materials

Obtaining unique materials	The CA (CMA activator) is synthesize in our laboratory. We have stated in page 23 "CA; available in small amounts upon request from the investigators laboratory"

Antibodies

Antibodies used	Antibody description indicating source, clone and dilution used is stated in Page 19-20..
Validation	Page 20 states: All antibodies used in this study were from commercial sources and were validated following the multiple dilution method and where available through the use of cell lines or tissues from animals knock-out for the antigen.

Eukaryotic cell lines

Policy information about cell lines

Cell line source(s)	Page 21 states: NIH3T3 used in this study were obtained from the American Type Culture Collection

Authentication	Page 21 states: [NIH3T3 cells] were validated by genomic PCR.
Mycoplasma contamination	Page 21 states: All the cells lines were tested for mycoplasma contamination using DNA staining protocol with Hoechst 33258 dye.
Commonly misidentified lines (See ICLAC register)	N/A

Animals and other organisms

Policy information about studies involving animals; ARRIVE guidelines recommended for reporting animal research

Laboratory animals	Page 19 states: Except where indicated, mice used for this study were FVB, males and females with ages ranging from 4-6 months of age. C57BL/6 KFERQ-Dendra mice were generated by back-crossing FVB KFERQ-Dendra mice with wild type C57BL/6 mice for 8 generations.
Wild animals	N/A
Field-collected samples	N/A
